# Effect on signal-to-noise ratio of splitting the continuous contacts of cuff electrodes into smaller recording areas

**DOI:** 10.1186/1743-0003-10-22

**Published:** 2013-02-21

**Authors:** Max Ortiz-Catalan, Jorge Marin-Millan, Jean Delbeke, Bo Håkansson, Rickard Brånemark

**Affiliations:** 1 Department of Signals and Systems, Biomedical Engineering Division, Chalmers, University of Technology, Gothenburg, Sweden; 2 Centre of Orthopaedic Osseointegration, Department of Orthopaedics, Sahlgrenska University Hospital, Gothenburg, Sweden; 3 School of Medicine (MD), Institute of Neuroscience (SSS/IoNS/COSY), Université Catholique de Louvain, Brussels, Belgium

**Keywords:** Cuff electrodes, Neural recordings, Neural prostheses

## Abstract

**Background:**

Cuff electrodes have been widely used chronically in different clinical applications. This neural interface has been dominantly used for nerve stimulation while interfering noise is the major issue when employed for recording purposes. Advancements have been made in rejecting extra-neural interference by using continuous ring contacts in tripolar topologies. Ring contacts provide an average of the neural activity, and thus reduce the information retrieved. Splitting these contacts into smaller recording areas could potentially increase the information content. In this study, we investigate the impact of such discretization on the Signal-to-Noise Ratio (SNR). The effect of contacts positioning and an additional short circuited pair of electrodes were also addressed.

**Methods:**

Different recording configurations using ring, dot, and a mixed of both contacts were studied *in vitro* in a frog model. An interfering signal was induced in the medium to simulate myoelectric noise. The experimental setup was design in such a way that the only difference between recordings was the configuration used. The inter-session experimental differences were taken care of by a common configuration that allowed normalization between electrode designs.

**Results:**

It was found that splitting all contacts into small recording areas had negative effects on noise rejection. However, if this is only applied to the central contact creating a mixed tripole configuration, a considerable and statistically significant improvement was observed. Moreover, the signal to noise ratio was equal or larger than what can be achieved with the best known configuration, namely the short circuited tripole. This suggests that for recording purposes, any tripole topology would benefit from splitting the central contact into one or more discrete contacts.

**Conclusions:**

Our results showed that a mixed tripole configuration performs better than the configuration including only ring contacts. Therefore, splitting the central ring contact of a cuff electrode into a number of dot contacts not only provides additional information but also an improved SNR. In addition, the effect of an additional pair of short circuited electrodes and the “end effect” observed with the presented method are in line with previous findings by other authors.

## Introduction

Cuff electrodes have been widely used in neuroprosthetics with different applications such as footdrop correction by stimulation of the peroneal nerve in patients with hemiplegia [1]; stimulation of the median and ulnar nerves for pain control [2]; treatment of obstructive sleep apnea by stimulation of the hypoglossal nerve [3]; and stimulation of the optic nerve in blind patients as part of a visual prosthesis [4].

Stimulation has been the main clinical application of cuff electrodes, with few exceptions such as recordings from the sural nerve for footdrop correction [5], and from the digital nerve for force-controlled hand-grasping [6]. The information provided by these recordings, however, has only been used in an “on/off” manner. In an animal model, offline discrimination between two to three different stimuli using a single channel cuff electrode has been demonstrated [7]. The signal to noise ratio (SNR), however, was reported as the main limiting factor for the classification performance, even in the absence of myoelectric interference which is known to be the major issue in cuff recordings [7].

In this study, we investigate different cuff configurations that could potentially increase the amount of information retrieved, and at the same time, preserve an acceptable SNR taking into account the main noise sources. When using cuff electrodes, the SNR depends on different factors such as the cuff geometry [8] and closure [9]; the recording configuration [10-12]; and the amplification electronics [13-17]. The latter has been approached in different ways, such as introducing audio transformers to match impedance, reduce the common noise (e.g. power lines), and as a passive pre-amplification [13,15]. Our study was focused on the recording contacts configuration in order to identify the best performing topologies.

**Figure 1:**
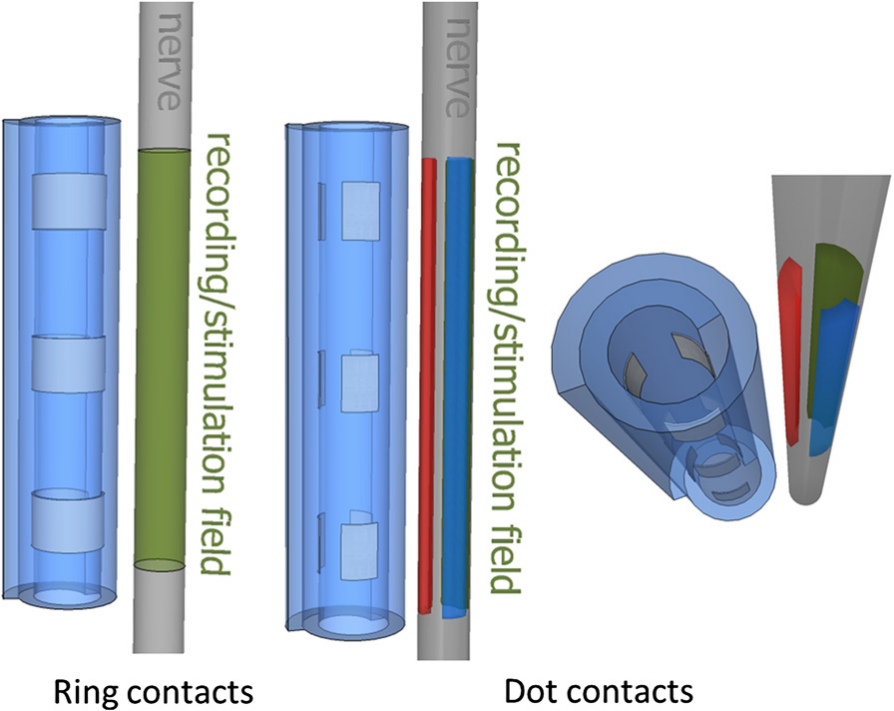
**Recording field with ring and dot contacts. **Two different cuff electrode designs using ring and discrete dot contacts are sketched. Ring contacts provide an average of the circumferential activity, while in the dot design splits this average theoretically providing more information. The split recording/stimulation fields are ideally constrained for illustration purposes only. Signals traveling in a volume conductor are expected to produce considerable crosstalk in adjacent contacts.

Most clinical implementations of cuff electrodes, as well as those for research purpose, have used continuous or so-called “ring” contacts [5,6,18-20]. These circumferentially continuous contacts record an average of the compound action potentials (CAPs) around the periphery of the nerve. A discrete (or “dot”) configuration, however, would potentially yield more information than continuous rings by decomposing the average of the field in a number of subsectors, as ideally illustrated in Figure 1. This idea has been exploited with different multi-contact cuff electrode designs, together with algorithms for extracting the fascicular source signals [21-26]. These have employed configurations with only dots [21-23], or a mixed of dot and ring contacts [24-26], where the impact on the contact type has not been addressed.

Dot configurations have been successfully employed for stimulation purposes [27-30]. It has been shown that dot contacts can be advantageous while using steering currents to better localize the stimulation [28]. In humans, selective activation of upper limb muscles has been shown feasible intraoperatively using this type of contacts [30].

Although the clinical use of dot contacts for recordings purposes is limited, feasibility has been shown in long-term (>2 years) selective recordings using multi-contact cuff electrodes in canines [21], where Rozman *et al.* used 4 out of 11 possible tripolar montages (3 bands of 11 contacts) to directly discriminate afferent signals from 2 muscles (1 source to 1 channel). Using statistical models, Cheng *et al.* achieved activity discrimination of the tibial and peroneal nerves in recordings obtained in the proximal sciatic nerve of rabbits (2 bands of 4 contacts) [22]. More recently, Zariffa *et al.* increased the number of contacts to 56 (7 bands of 8 contacts) to proximally discriminate the tibial, peroneal, and sural nerves using an experimental leadfield approach [23]. Although relatively satisfactory in offline signal discrimination under controlled environments, the SNR was admittedly low [21], which would hinder stability in a clinical implementation.

The flat interface nerve electrode (FINE) [31], a cuff-like neural interface, has been used in a combination of ring and dot contacts to selectively record signals from multifasciculated nerves in canines [32]. Spatial filtering or beamforming algorithms have been used to extract neural information with considerable crosstalk from this multi-contact electrode [25,26]. The contacts selection, however, has not been motivated.

**Figure 2:**
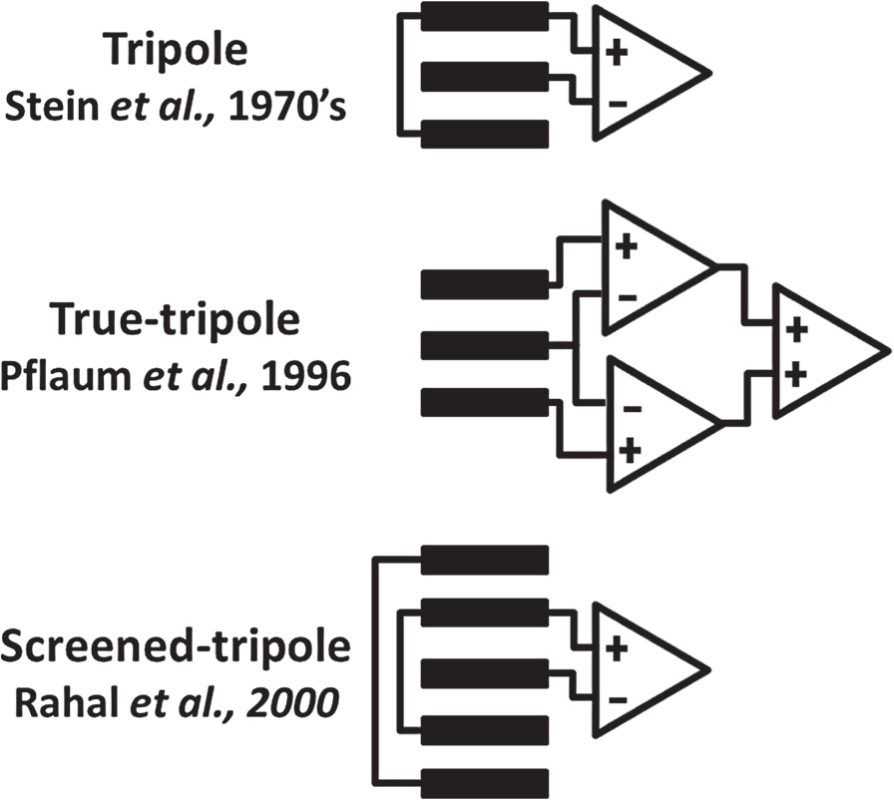
**Cuff recording configurations. **The top inset is the first introduced tripolar configuration by Stein *et al. *[35,36]; next, the true-tripole by Pflaum *et al. *[11], and at the bottom, the screened-tripole presented by Rahal *et al. *[12].

Cuff electrodes are currently the most commonly used clinical neural interface, while FINE is still proving its safety and efficacy with promising results. These multi-contact electrodes have the potential to improve the controllability of neuroprostheses and artificial limbs [33]. The optimal selection of contact type and recording configuration thus remains as a relevant question. When recorded with cuff electrodes, the amplitude of neuroelectric signals (NES) is in the order of a few *μ*Vs [34]. These low amplitudes make them highly susceptible to interference, especially from myoelectric signals (MES) which are generated nearby in the surrounding muscles with typical amplitudes three orders of magnitude larger. The tripolar recording cuff electrode introduced in the 1970’s by Stein *et al.* was the first considerable achievement in rejecting external noise such as MES and stimulation artifacts (SAs) [35,36]. In this context, a tripolar configuration means a differential measurement between two short-circuited end contacts and a central contact (Figure 2). This configuration was later called *quasi-tripolar* by Pflaum *et al.*, and a double differential configuration was proposed as a *true-tripole*[11] (Figure 2). Although the true-tripolar configurations yielded a 7% improvement over the quasi-tripole, authors still use the term tripolar as Stein *et al.* did [8,10,12,37-39]. Rahal *et al.* made the latest improvement to the tripole by adding an additional pair of short-circuited contacts [12]. This configuration is known as the screened-tripole or short-circuited tripole [10,39] (Figure 2). It is noteworthy, that in multi-contact cuff electrodes several tripolar configurations can be applied together. It has been calculated that multiple ring contacts in a double differential amplifier arrangement (true-tripoles) would be more sensitive to slow action potentials (20 m/s) than a single tripole [20]. Moreover, additional electronics can be used on top of the true-tripole to compensate for the cuff imbalance as suggested by Triantis *et al.* as the adaptive-tripole [17].

The tripolar configuration allows a noise field reduction by short-circuiting the end electrodes [12]. It has been suggested that the interfering signal picked up by the end electrodes travels through the short-circuited path rather than in the cuff, thus, reducing the interference [35]. The efficiency of the tripole, and its later modifications, resides in the linearization of the extra-fascicular noise field by the physical confinement due to the cuff itself [11,16]. The main purpose of this study was to investigate if the screening properties of the tripole are degraded by splitting the ring contacts into single dots. At the same time, we extended the investigation to study the effect of displacing the recording contacts inwards the cuff (the “end effect”), as previously analyzed theoretically [8,12,39], and tested in an animal model [10].

## Methods

### Experimental settings

In order to compare how different recording configurations affect the SNR, an experiment was designed where the sources of noise and other environmental factors were kept constant. The only variable was the assignment of the electrode contacts to the differential amplifier inputs.

**Figure 3:**
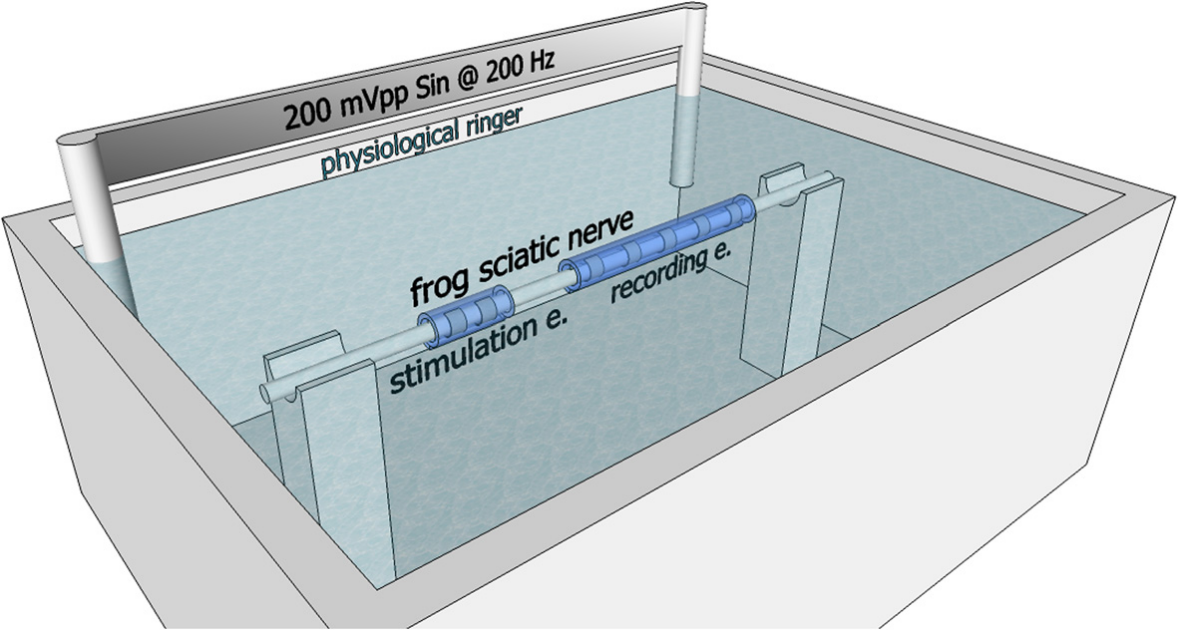
**Experimental setup. **Recording and stimulation electrodes were wrapped around the explanted sciatic nerve of a frog. These were submerged in a physiological Ringer solution. Two electrodes were placed at opposite ends of the container and powered with a sinusoidal signal of 200 *mV*_*pp *_at a frequency of 200 *Hz*. The tied extremities of the nerve (not shown) were attached to maintain the nerve extended and submerged.

This study has been approved by the committee for ethical use of animals of the faculty of Medicine of the Universit catholique de Louvain. Experiments were conducted *in vitro*, on the excised sciatic nerves of 8 adult frogs (Rana catesbeiana). After spinalization of the animals, the sciatic nerves were carefully dissected relatively distally in order to avoid branching. Both ends of a selected 40 *mm *long segment were ligatured with a silk suture. The corresponding length of the sciatic nerve was then cut out a little further to yield a free nerve stretch of approximately 1 *mm *diameter. This was transferred to the recording chamber, placed as shown in Figure 3 and attached by its sutured extremities.

The nerve was entirely submerged in physiological Ringer solution at 24° C (±2° C). In order to simulate the noise induced by surrounding muscles, silver electrodes were placed in the solution approximately 55 *mm* apart, in opposite corners of the container. These were driven by a 200 *mV*_*pp *_sinusoidal signal at 200 *Hz*. This noise is further referred to as the *simulated myoelectric signal *(SMES). The 200 *Hz *frequency corresponds to a normal peak frequency of myoelectric signals [40-42]. The 200 *mV*_*pp *_amplitude was selected for being high enough to appear in the recordings of all configurations, thus, allowing comparisons to be made. A drawing of the experiment setup is given in Figure 3. The cuff electrodes were finally wrapped around the nerve, with the stimulation electrode near the end of the nerve that would have been proximal in the body.

The stimulation of the nerve was performed with an asymmetric charge balanced pulse (Lilli pulse). In this experiment, however, at the stimulating contact (the one closest to the recording electrodes), the first phase was the anodic charge compensating pulse (70 *μ*A amplitude and 250 *μ*s duration) while the second phase represented the actual cathodic phase (-350 *μ*A and 50 *μ*s respectively). This pulse shape was used in order to ease the identification of the SA and the actual CAP.

A single experiment consisted in the preparation of the sciatic nerve; placement of the recording and stimulation electrodes; submersion of the nerve in physiological Ringer solution; fixation of the electrodes inducing the SMES; fixation of the reference electrode in a corner opposite to the SMES (not illustrated). The experiments were performed inside a Faraday cage and the recordings were amplified 100 times with a low pass filter at 3 *kHz *(Tektronix ADA400A), and digitalized at 10 *kHz*. For each configuration of a single experiment, 6 recordings were obtained and averaged for each of the three signals: the CAP, the SA and the SMES. Peak to peak amplitudes were measured as the study dependent variables. The complete experiment was repeated 16 times in different nerves and with different electrodes.

Since this study is about comparing the amplitude of the CAP against SA and SMES, it was important to make sure the nerve remained healthy for the whole duration of each experiment. For that purpose, the nerve conduction velocity (CV) and the CAPs’ amplitude were recorded at the onset and end of each experiment, and then compared. The CV and CAPs were obtained from two simultaneous bipolar recordings derived from both nerve end pair of contacts. No experiment had to be rejected on the basis of significant nerve degradation as reported in the results section.

### Electrode designs

All the spiral cuff electrodes [43] used here were 26 *mm *long with an inner diameter of 1 *mm*. The platinum contacts were longitudinally spaced by 5 *mm *(center to center) and the extreme ones were at 3 *mm *from the cuff ends (center to edge). Two types of contacts were used, named respectively the *dot *and the *ring *contacts. The dot contacts were made of 1x1 *mm *squares embedded in the silicone rubber with a round opening of 500 *μm *diameter facing the nerve. The ring contacts were made of 1x3 *mm* platinum foils with 3 openings of 500 *μm *distributed around the nerve periphery.

**Figure 4:**
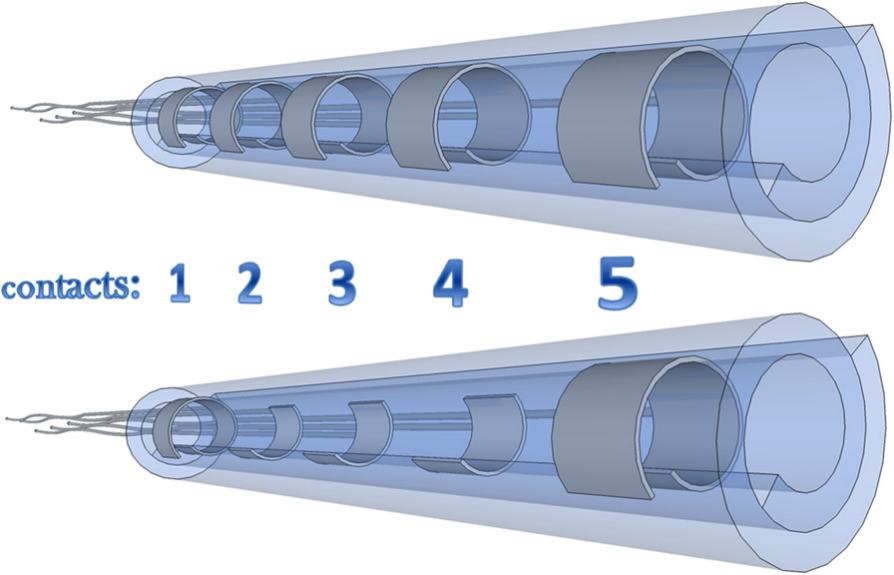
**Cuff electrode designs. **Illustrative spiral cuff electrodes in *ring* and *mixed *design used in this study. Top and bottom insets respectively. These were 26 mm long cuffs, 1 mm inner diameter, 5 mm inter-contact distance (center to center), and 3 mm contact distance to the edge. These designs allowed recordings in *ring *(r), *dot *(d) and *mixed *(m) configurations.

Two types of spiral cuff electrode designs respectively labeled *ring *and *mixed *design, were explored (see Figure 4). These allowed to test different recording configurations labeled as *ring *(r), *dot *(d) and *mixed *(m). The *mixed* label refers to the case where ring and dot contacts were connected to the amplifier. If only one type of contact was used, the configuration was named accordingly.

The same stimulation electrode was used in all experiments. It consisted of a 5 *mm* long spiral cuff [43] with 2 ring contacts of 1x3 *mm *separated from each other and from the cuff ends by 1 *mm *space. The opening windows were similar to the ones described for the ring contacts.

**Figure 5:**
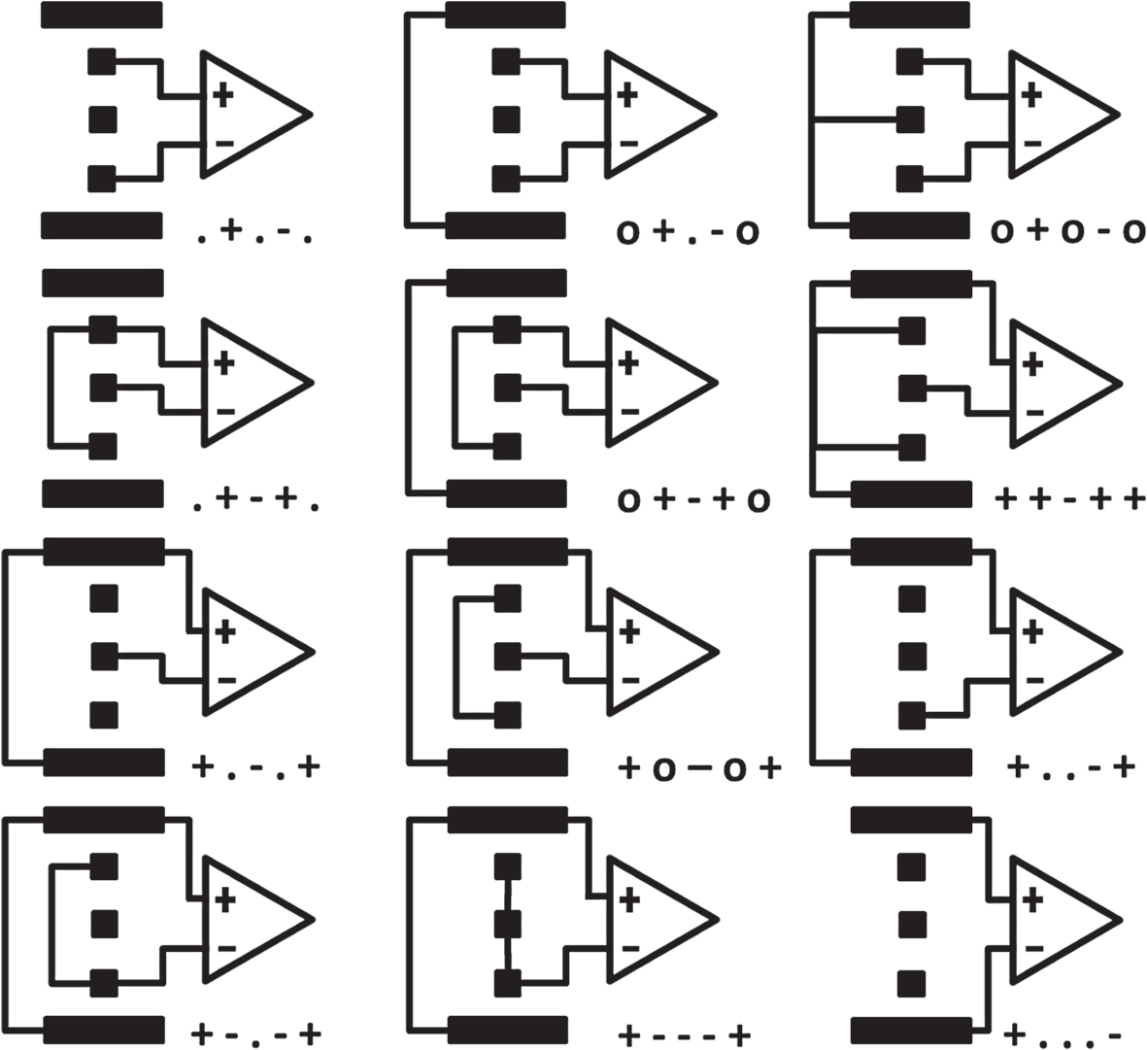
**Recording contact configurations for the mixed design. **The connection of each contact is represented by: “.”, if there was no connection; “o”, for short-circuited terminals; and “+” or “-”, indicate the differential amplifier terminal. Dot contacts are represented by squares and ring contacts by strips.

The following nomenclature was used to identify each recording configuration. Each of the 5 electrode contacts is represented by a single printing sign. These are ordered sequentially as the contacts they represent, starting with the one furthest away from the stimulation. When a contact is not connected, it is represented by “.” while “o” stands for contacts shorted together; “+” or “-”, indicate the corresponding input terminal of the differential amplifier. Finally, a prefix “r”, “d” or “m” is added in front of the string above when differentiation between the ring, dot and mixed configurations respectively is required. The 12 mixed recording configurations are represented in Figure 5. The corresponding 12 ring configurations would yield a similar representation except that the 3 middle dots would be replaced by a rectangle to represent a ring.

### Data analysis

The measured CAP, SA and SMES values vary randomly between different experiments due to slight differences in the experimental setup, such as the cuff fitting around the nerve; the nerve dimensions and physiological status; the nerve conduction velocity; the precise placement of the electrodes inducing SMES; and the separation of the stimulation and recording electrodes. However, once the experimental setup is completed, the only intra-study difference between the recording configurations is the connection of the electrode contacts to the differential amplifier. Therefore, we choose to focus on the improvement ratios (IRs), a concept developed by Andreasen and Struijk to compare different recording configurations [10,39].

In this application, the IRs can be defined as follows:

If *i* and *j* refer to the CAPs of two configurations from the same session, the resulting ratio (rCAP) is represented by:

(1) rCAP=CAPj/CAPi

Similarly, we quantify the SMES ratio as rSMES = SMES*j*/SMES*i*, and the SA ratio as rSA = SA*j*/SA*i*. The CAP, SMES and SA correspond to the peak to peak values.

The SNR corresponding to SMES and SA are represented by:

(2) $$\begin{array}{ll} \text{SNR(SA)} & =\text{CAP}/\text{SA}\; \end{array}$$

(3) $$\begin{array}{ll} \text{SNR(SMES)} & =\text{CAP}/\text{SMES}\; \end{array}$$

Finally, the performance between configurations was evaluated using the improvement ratio (IR) calculated for each of the two types of SNRs:

(4) $$\begin{array}{ll} \text{IR(SA)} & =\text{SNR(SA)}j/\text{SNR(SA)}i\; \end{array}$$

(5) $$\begin{array}{ll} \text{IR(SMES)} & =\text{SNR(SMES)}j/\text{SNR(SMES)}i\; \end{array}$$

The non-parametric, Wilcoxon signed rank test, was used to evaluate the significance of pair-wise differences between configurations in the same electrode design and experiment or session (same nerve, electrode, and environmental conditions). A different non-parametric test, the Mann Whitney U-test, was required to compare configurations from different electrode designs since the pair-wise relationship is non-existing in this case. When comparing recordings from different electrode designs and therefore, different sessions, the effects of the experimental setup were accounted for by using recordings from one of the configurations used in the same experiment as a normalization parameter. In our case, this configuration was the bipolar recording between contact 1 and 5 (+...-), since it is identical in both electrode designs. The significance level used for both statistical tests was p = 0.05.

## Results

**Figure 6:**
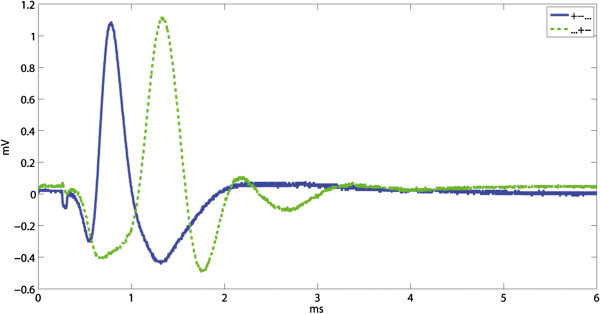
**Example of compound action potentials. **Example of compound action potentials (CAPs) recorded between the end contacts, (+-...) and (...+-), as used to measure the conduction velocity (CV). The CV and CAPs’ amplitudes were repeatedly measured at the onset and at completion of the experiment in order to exclude sessions where nerve degradation occurred.

The nerve CV and CAPs’ amplitudes were measured from simultaneous recordings at the extreme electrodes (+-...) and (...+-), corresponding to a distance of 15 *mm* and yielding responses as shown in Figure 6. The average nerve CV was 25.54 *m/s* (±4.64) with a reduction of 1.20% (±3.99%). The reduction in CAP was in average 1.70% (±9.31%) between onset and completion of each experiment. Both CV and CAP did not always decrease, perhaps due to small metabolic shifts in the artificial medium. These changes, however, remained very small in comparison with the statistically significant results given hereafter.

**Figure 7:**
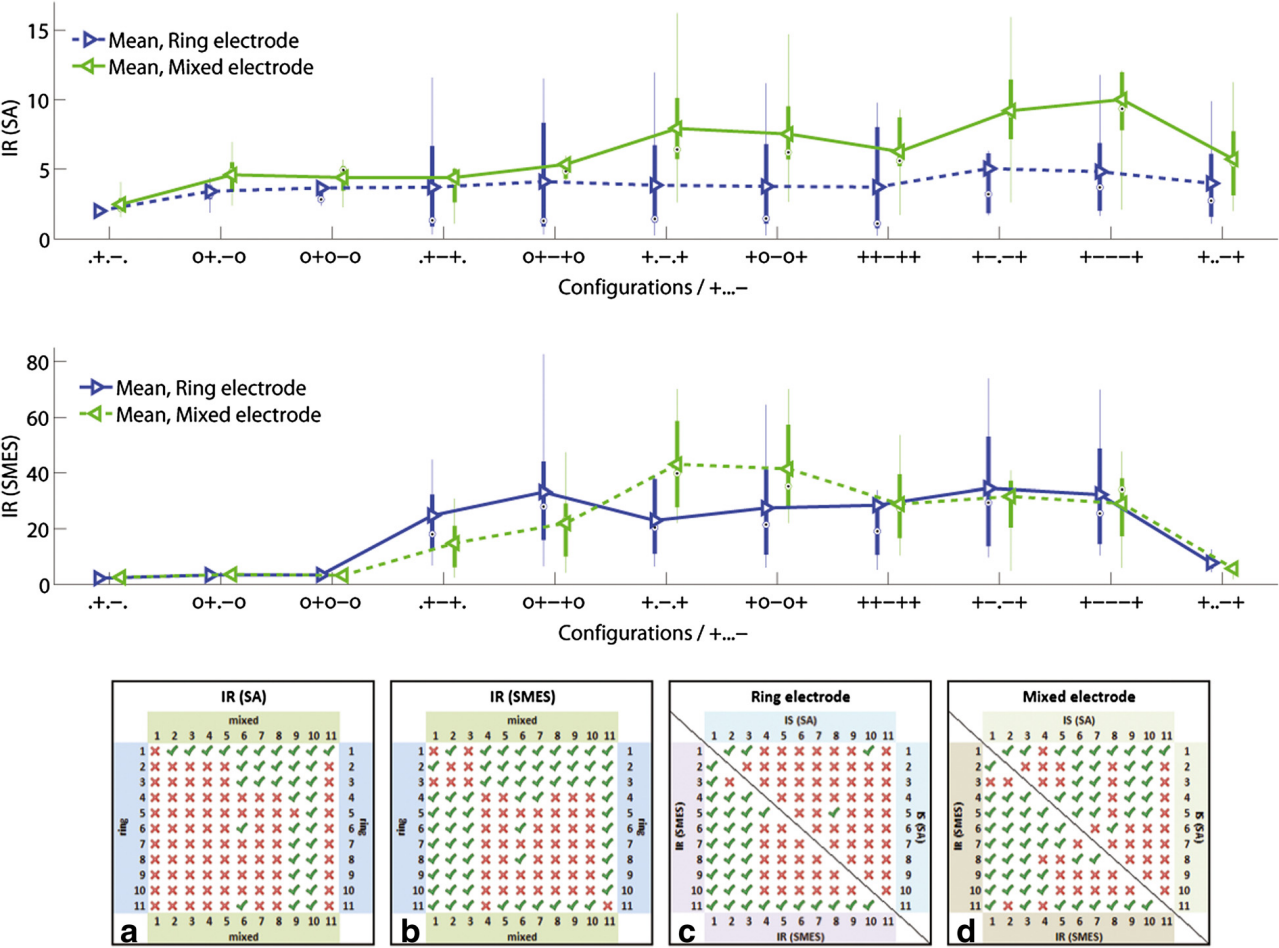
**Improvement ratios (IR). **The IR(SA) and IR(SMES) results are presented in box plots where the central mark represents the median value; the edges of the box are the 25th and 75th percentiles; the whiskers give the range of data values; and laterally pointing triangles represent average values. The results for ring and mixed designs are represented in blue and green colors respectively. The IRs were calculated against the outer bipolar configuration, +...-., since this is common to both electrode designs. The statistical significance (*p *< 0.05) between the configurations in ring and mixed electrodes are shown in **a)** for SA and **b) **for SMES, as well as between inter-electrode configurations in **c) **for the ring electrode, and **d) **for the mixed electrode. The configuration numbers correspond to the order in which they are presented in the box plots.

In order to allow a comparison between experiments, results are presented in IRs referred to the outer bipolar configuration (+...-) of the same session. This can be observed in Figure 7 where the overall IRs for both SNRs (SMES and SA) are presented.

### The effect of reducing the tripolar length by moving the contacts inwards

**Figure 8:**
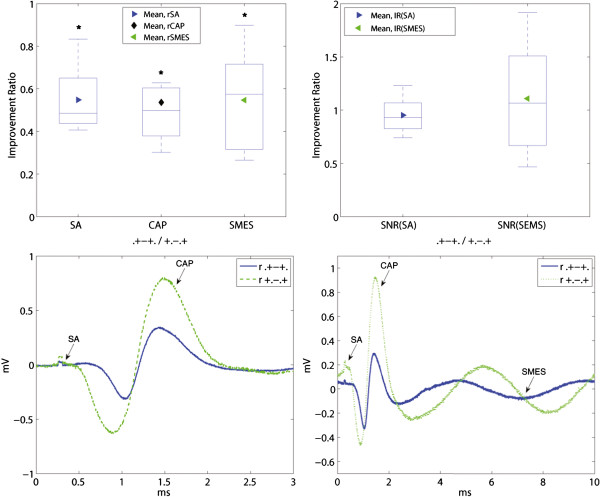
**Effect of moving the end tripolar contacts inwards the cuff. **The rCAP and both interfering signals rSA and rSMES are shown on the upper-left inset. The resulting IRs of both SNRs are plotted in the upper-right inset. The results are presented in box plots where the central mark represents the median value; the edges of the box are the 25th and 75th percentiles; the whiskers give the range of data values; laterally pointing triangles represent average values. An asterisk is used to show the statistically significant IR (*p *< 0.05). The bottom illustrative recordings show the “end effect” for the 5 *mm *inwards contact displacement. The noise reduction was very close to that of the compound action potential (CAP) thus having a small impact in SNR. The stimulation artifacts (SAs) and CAPs appear chronologically in the lower-left inset without the induced simulated myoelectric signal (SMES). Recordings from both configurations were obtained during the same session.

**Figure 9:**
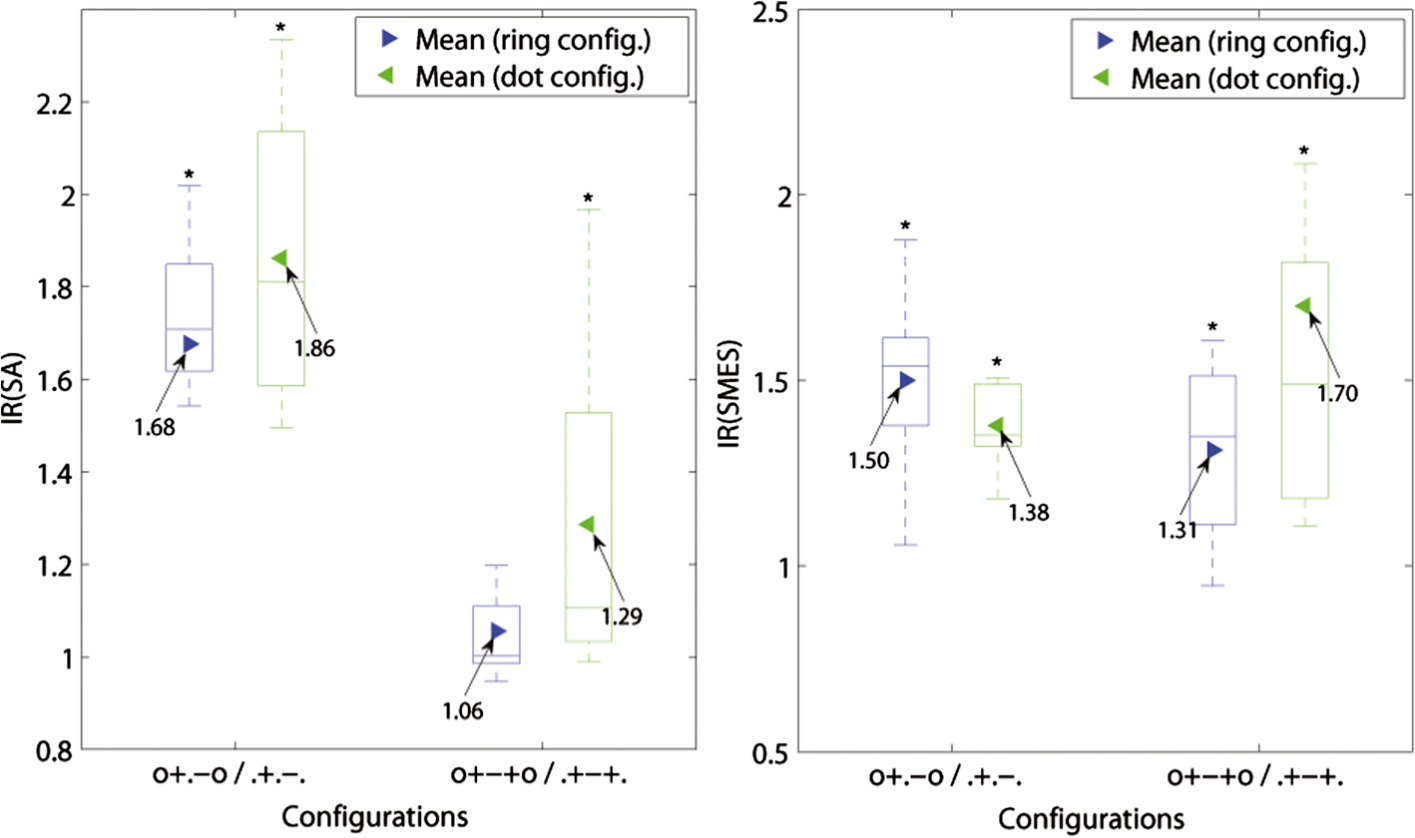
**Effect of an additional short-circuited pair of electrodes on bipolar and tripolar configurations. **The IRs are presented in box plots where the central mark represents the median value; the edges of the box are the 25th and 75th percentiles; the whiskers give the range of data values; laterally pointing triangles represent average values. An asterisk is used to show the statistically significant IR (*p* < 0.05).

The effect of moving the end tripolar contacts inwards the cuff was evaluated by computing the IRs for the tripolar ring configurations (r,.+-+./+.-.+), Figure 8. The mixed electrode is not used in this comparison since the change from ring to dot contacts would be confounding. The amplitude of the interfering noise was reduced by rSA = 0.55 (r,.+-+./+.-.+) and rSMES = 0.56 (r,.+-+./+.-.+), both statistically significant. The amplitude of the CAP, however, was also reduced by rCAP = 0.54 (r,.+-+./+.-.+), p<0.05, which ultimately generated a negligible impact in SNR with IR(SA) = 0.96 and IR(SMES) = 1.04, both without statistical significance. An example of the recorded signals is shown in Figure 9.

The end contacts displacement corresponds to a 50% reduction of the tripolar length which incidentally resulted in around 50% reduction of all the signals recorded, as intuitively expected in a proportionally linear relationship but not observed by other authors [10,39].

### Effect of an additional short-circuited pair of electrodes

Short-circuiting a pair of ring electrodes in the cuff ends proved to increase the SNR in both bipolar and tripolar recordings, for both ring and dot configurations (see Figure 9). The SNR of bipolar ring recordings was increased on average by IR(SA) = 1.68 (r, 0+.-0/.+.-.), and IR(SMES) = 1.50 (r, 0+.-0/.+.-.), both with statistical significance. The effect on the tripolar configuration was smaller with IR(SA) = 1.06 (r, 0+-+0/.+-+.), and IR(SMES) = 1.31 (r, 0+-+0/.+-+.). Only the latter reached statistical significance.

**Figure 10:**
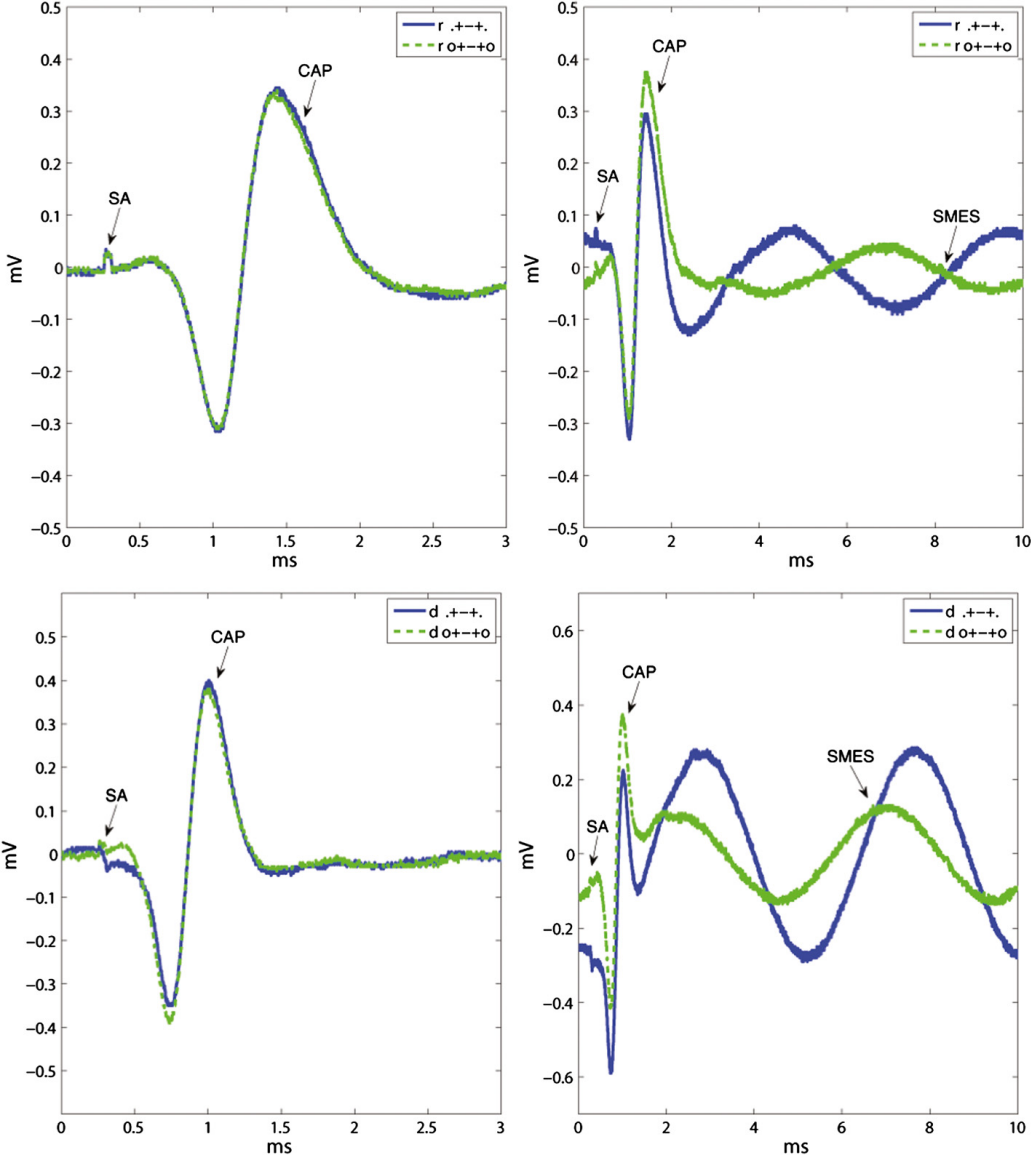
**Recording examples of effect of an additional short-circuited pair of electrodes.** Illustrative recordings showing examples of the effect of an additional pair of short-circuited electrodes (o...o). The upper and lower insets correspond to the dot and ring configurations respectively. The stimulation artifacts (SAs) and compound action potentials (CAPs) appear chronologically in the left insets without the induced simulated myoelectric signal (SMES). Recordings from both configurations in each inset were obtained during the same session.

The dot configurations showed an average SNR improvement in bipolar recordings with IR(SA) = 1.86 (d, 0+.-0/.+.-.) and IR(SMES) = 1.38 (d, 0+.-0/.+.-.), as well as in tripolar recordings with IR(SA) = 1.29 (d, 0+-+0/.+-+.) and IR(SMES) = 1.70 (d, 0+-+0/.+-+.), all statistically significant. Although the impact of additional short-circuited electrodes was on average higher for the dot configurations, no statistically significant difference was found between ring and dot configurations. An example of these recordings is given in Figure 10.

### The effect of splitting the ring contacts

We found important but statistically non-significant differences between SNRs of ring and dot configurations in bipolar recordings (.+.-.) with IR(SA) = 0.81 (r.+.-./d.+.-.), and IR(SMES) = 0.87 (r.+.-./d.+.-.). Tripolar recordings (.+-+.) also yielded rather large differences with IR(SA) = 0.84 (r.+-+./d.+-+.), and IR(SMES) = 1.67 (r.+-+./d.+-+.), however, again without statistical significance.

**Figure 11:**
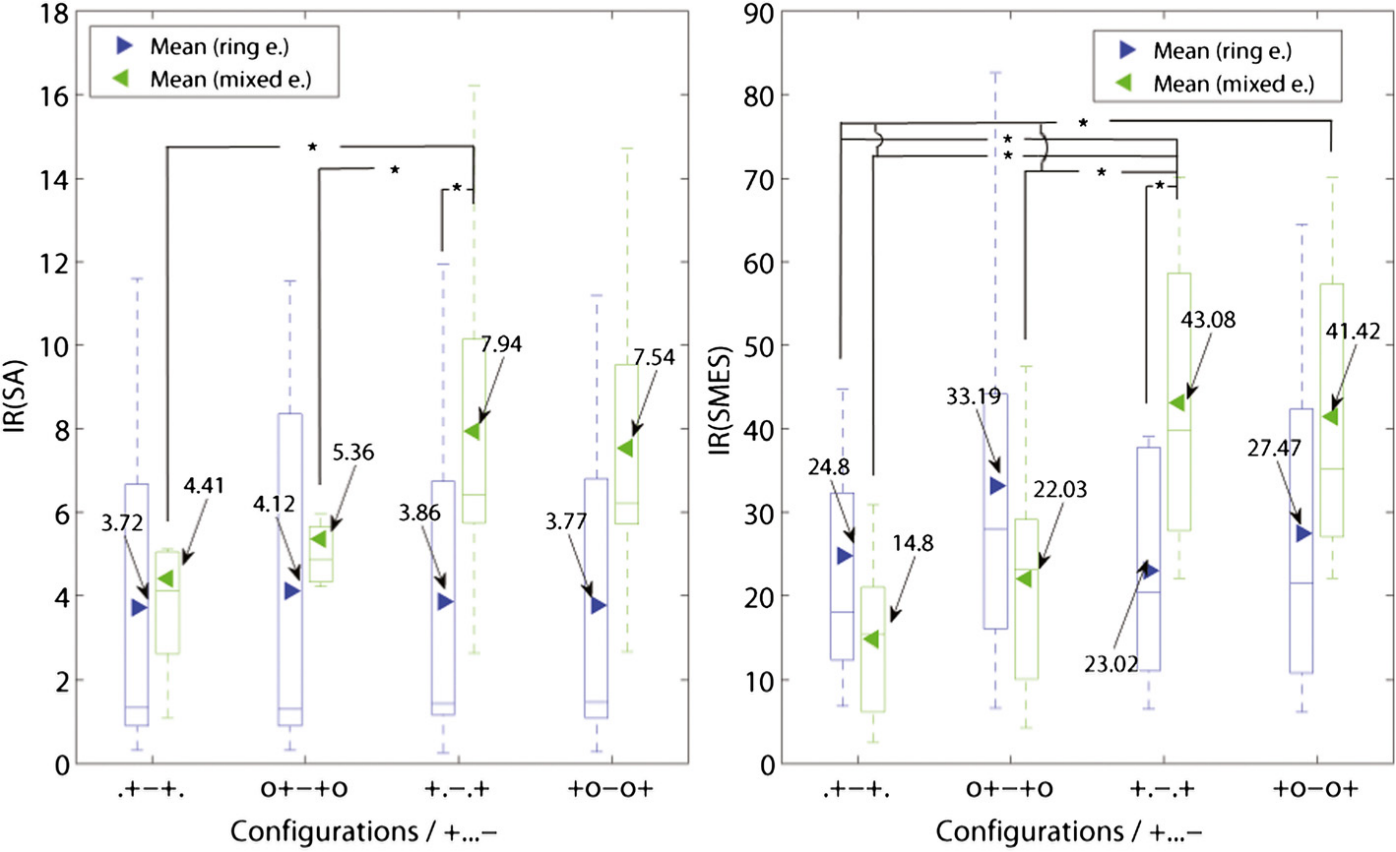
**Comparison between ring, dot and mixed configurations. **Comparison between ring (left-side boxes) and mixed (right-side boxes) designs in tripoles, short-circuited or not: (.+-+.), (o+-+o), (+.-.+) and (+o-o+). The IRs are referred to the corresponding outer bipolar configuration, (+...-). An asterisk is used to show the statistically significant IR (*p *< 0.05), for further details see Figure 7.

An alternative to complete dot configurations is a combination between ring and dot contacts. These mixed configurations outperformed the ring configurations in both SNRs (SA and SMES) as presented in Figure 11. In tripolar recordings (+.-.+), the improvement of mixed over ring configurations was IR(SA) = 2.06 (m/r, +.-.+) and IR(SMES) = 1.87 (m/r, +.-.+), both statistically significant. A variation of the latter configuration with an additional inner pair of short-circuited electrodes (+o-o+) showed an IR(SA) = 2.00 (m/r, +o-o+) and IR(SMES) = 1.51 (m/r, +o-o+). The latter IR did not reach significance.

The configurations with the best SNR against SMES were the ring short-circuited tripole (ro+-+o) and the mixed tripole (m+.-.+), for ring and mixed electrode designs respectively. The differences between these two configurations yielded an IR(SMES) = 1.30 (m+.-.+/ro+-+o) without reaching statistically significance. The improvement of SNR rejecting SA was higher with IR(SA) = 1.93 (sd+.-.+/ro+-+o), however still not statistically significant. Figure 11 shows the IR of the latter configurations which have the best SNR(SMES).

**Figure 12:**
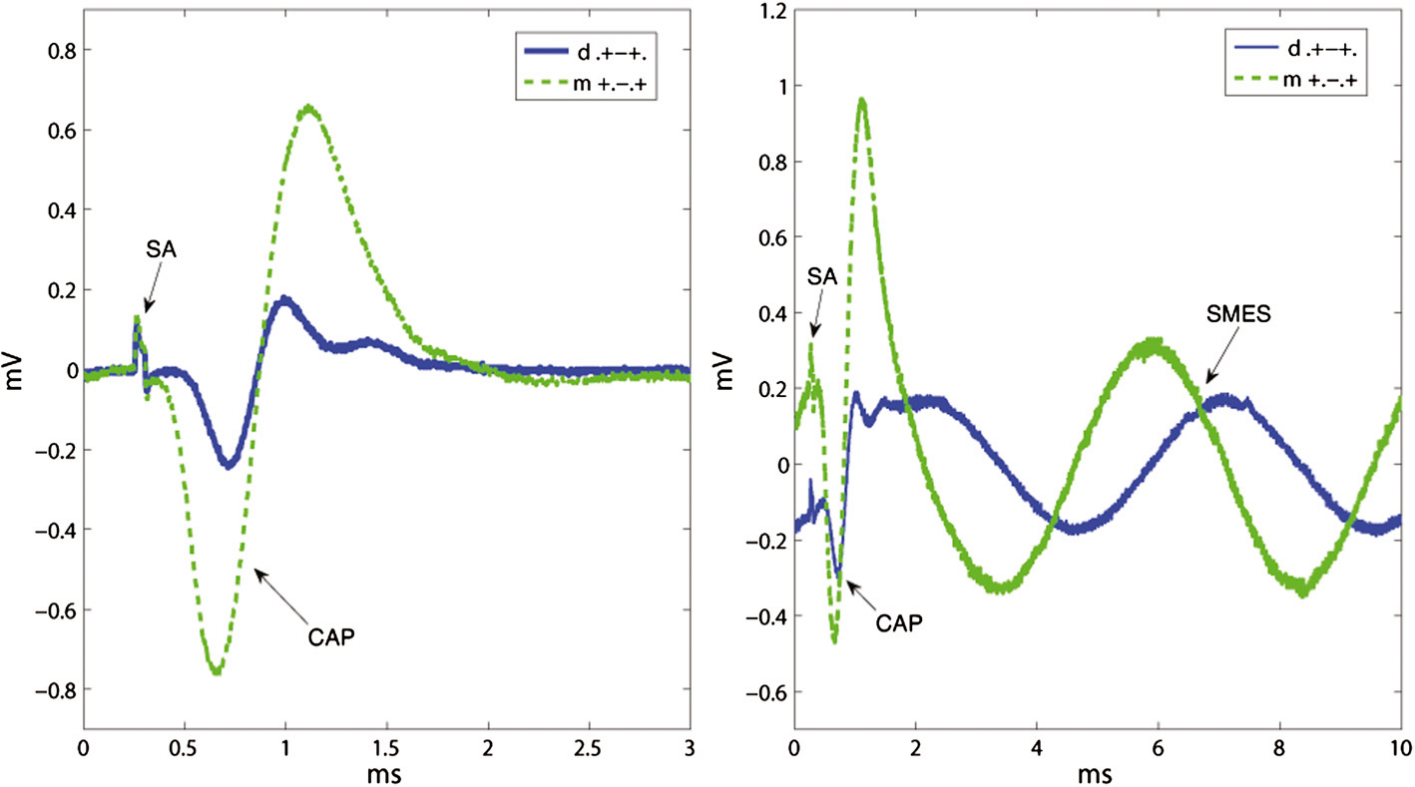
**Recording examples that compare dot and mixed configurations. **The stimulation artifacts (SAs) and compound action potentials (CAPs) appear chronologically in the left inset without the induced simulated myoelectric signal (SMES). Recordings from both configurations were obtained during the same session. Since the “end effect” was found negligible for 5 *mm *inwards contact displacements in our experiments, the difference in SNR can be mostly attributed to the change between ring and dot outer contacts. This resulted in an average IR(SMES) = 2.91 (m+.-.+/d.+-+.) and IR(SA) = 1.80 (m+.-.+/r.+-+.).

Furthermore, both mixed configurations, (+.-.+) and (+o-o+), reached a statistically significant level of improvement for the SNR(SMES) over the dot tripolar (d.+-+.), with IR(SMES) = 2.91 (m+.-.+/d.+-+.) and IR(SMES) = 2.80 (m+o-o+/d.+-+.) respectively. The improvements for SA were smaller with IR(SA) = 1.80 (m+.-.+/d.+-+.), and IR(SA) = 1.71 (m+o-o+/d.+-+.), but still statistically significant. An example of recordings with both dot and mixed tripoles is given in Figure 12. Moreover, the mixed tripolar montage (+.-.+) yielded a statistically significantly larger SNR than the short-circuited dot tripole (o+-+o) with IR(SMES) = 1.95 (m+.-.+/do+-+o) and IR(SA) = 1.48 (m+.-.+/do+-+o). As expected, these IRs were lower than the previous ones because in this case, the dot tripole benefits from an additional pair of short-circuited electrodes. Although the contact position is an extra variable in the latter comparisons, we observed no considerable change in SNRs due to this specific electrode displacement (5 *mm *inwards) as mentioned previously. Under the same rationale, we report the IRs against the ring tripole (r.+-+.). This was also statistically significant with an IR(SMES) = 1.74 (m+.-.+/r.+-+.) and IR(SMES) = 1.67 (m+o-o+/r.+-+.). The average improvement for SA was higher with IR(SA) = 2.14 (m+.-.+/r.+-+.) and IR(SA) = 2.03 (m+o-o+/r.+-+.), but nevertheless these IRs did not reach statistical significance.

**Figure 13:**
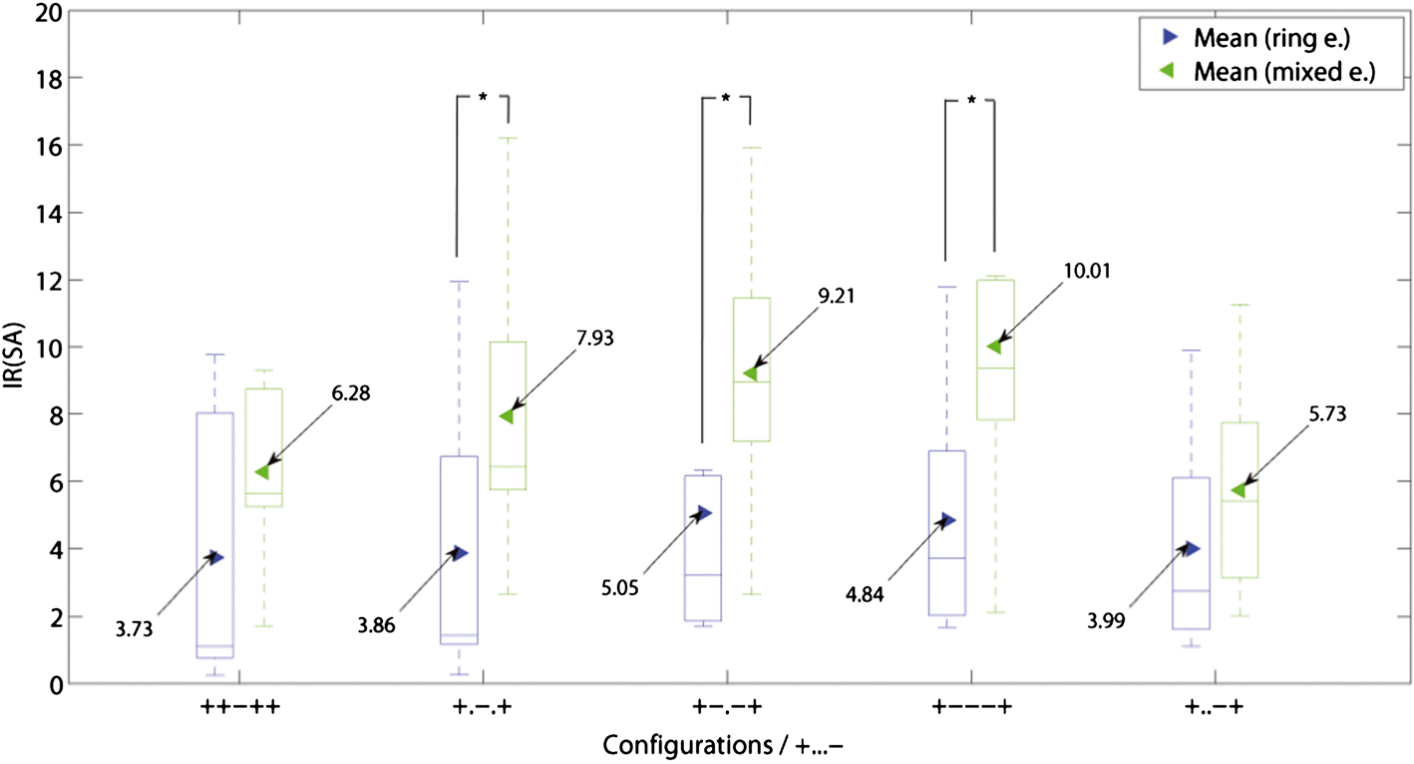
**Best configurations rejecting the stimulation artifact. **Different mixed configurations outperforming their counterparts with ring contacts in the rejection of the stimulation artifact (SA). An asterisk is used to show the statistically significant IR (*p *< 0.05) for the same configuration in different electrodes. For other statistically significance levels, please refer to the tables in Figure 7.

The SNR against SA was further improved using a different mixed configuration (see Figure 13). The mixed electrode design allows to simulate a larger recording area in the longitudinal direction by connecting the three central contacts and setting up a tripolar montage with the outer two (+—+). These mixed configurations yielded an improvement over their ring counterparts by IR(SA) = 2.05 (m/r, +.-.+), IR(SA) = 1.82 (m/r, +-.-+), and IR(SA) = 2.07 (m/r, +—+). These differences were statistically significant. Another possible configuration was to increase the area for the end contacts which turned out to be less advantageous in comparison with the previous configuration. It yielded a statistically non-significant IR(SA) = 1.68 (m/r, ++-++). All the latter comparisons were neither large nor significant for SMES.

Finally, the effect of asymmetry in SNR(SA) for the tripolar configuration can be observed in Figure 13. This effect was most important for the mixed configuration with IR(SA) = 1.38 (m, +.-.+/+..-+) in comparison with the ring configurations with IR(SA) = 0.96 (r, +.-.+/+..-+), although, both with non-statistical significance. The effect of asymmetry was also higher for the mixed configurations when evaluating for SMES with IR(SMES) = 7.51 (m, +.-.+/+..-+), against IR(SMES) = 2.93 (r, +.-.+/+..-+) of the ring tripole, both with statistical significance. The difference between the asymmetric tripoles, however, was not significant when comparing the ring and mixed designs (m/r, +..-+) with IR(SA) = 1.44 and IR(SMES) = 0.74.

## Discussion

### The effect of displacing the end tripolar contacts inwards the cuff

In agreement with our results, Andreasen and Struijk have reported a decrement in CAP and both interfering signals (SA and SEMG) when moving the contacts inwards the cuff. These authors also found a progressively more modest SNR increment when the contacts were moved further inside (the “end effect”). This mainly resulted from the fact that the noise reduction rate approximated that of the CAP. Taking this into account, they concluded that a cuff inwards placement of 14.8% relative to the cuff length was close to optimal. For their cuff of 27 *mm* length and 2 *mm *diameter, this meant a distance of 4 *mm *from the contact’s center to the cuff edge. We placed the end contacts of the tripole 30.7% inwards (.+-+.), and found no significant improvement in SNR when compared with a 9.6% inwards placement (+.-.+). However, there was a statistically significant reduction of CAP, SA, and SEMG. These reductions were close enough not to impact the SNRs significantly as expected from previous research [10]. This suggests that if signal amplitude only is considered, the optimal contact placement is between 10% and 30% inwards a 26 *mm *long cuff with 1 *mm* diameter. Note that reducing the cuff length increases the interference pick up close to the ends [8]. Combined with a reduction of the spatial extent of the recording tripole, the inwards displacement of the end contacts used in this study has a negligible effect on SNR which is relevant to the rest of the study. It is worthy of mentioning that this study was performed with a defined cuff length and increasing it together with the tripole width will improve the SNR. However, the cuff length is strongly limited by the anatomy and implantability requirements.

### Effect of an additional short-circuited pair of electrodes

Our results, as well as previous research, showed an increment in SNR when using an additional short-circuited pair of electrodes [10,39]. This improvement was observed in bipolar as well as tripolar recordings, and for both ring and dot configurations. This electrode feature resulted in a 68% (0+.-0/.+.-.) improvement for SA and 50% for SMES. In tripolar recordings (0+-+0/.+-+.), the results are clearly less with 6% improvement for SA and 31% for SMES. As previously observed in ring configurations [10], we thus found the effect of an additional short-circuited pair of electrodes to be higher in bipolar than in tripolar configurations. Andreasen and Struijk suggested that the larger improvement in bipolar recordings could be explained by considering that the tripolar configuration already includes a short-circuited pair of electrodes. Our results support this argument with the additional observation that the dot tripolar configuration, in which the shorted extremes do not form full rings, had higher IRs (0+-+0 /.+-+.) than tripoles whereby the shorted outer contacts are full rings, 22% (d/r) for SA and 30% (d/r) for SEMG. The latter suggests a negative impact on the noise field reduction as the extend of a contact around the nerve is reduced to a dot. The differences between these two configurations, however, were not statistically significant in our work.

The short-circuited tripole has been shown to be the best performing configuration [10,12,39], mainly because it benefits from the “end effect” and the additional short-circuited pair of electrodes [10,39]. The “end effect” known as the reduction of the interfering noise by moving the contacts inwards the cuff, is in our case not contributing to an improvement since the signal of interest (CAP) is also reduced in similar proportions. Therefore, it is worth noting that the observed improvement in SNR most likely corresponds to the additional short-circuited electrodes only.

As mentioned in the introduction, there are two types of tripolar montages: 1) tripolar, and 2) true-tripolar. These configurations reduce the effect of electrical sources located outside the cuff by using the average of the linearized field as a reference. The tripole reduces the externally induced field by short-circuiting it, which is not the case in true-tripole since it has all three contacts connected to the high input impedance of the amplifiers. This has to be taken into consideration while extrapolating these results to true-tripolar recordings.

### Effect of using dot contacts over ring contacts

Although the proposed mixed tripole (m+.-.+) yielded higher SNRs than the previously best known configuration, namely the ring short-circuited tripole (ro+-+o) [10,11,39] by 30% for SMES and 93% for SA, these differences do not reach statistical significance. These findings, however, suggest that an additional pair of short-circuited electrodes might not be necessary when employing a mixed tripole. Reduction of the required number of contacts could be a practical advantage of such scenario. Two interpretations can be considered here: 1) the reduction in noise rejection is compensated by an increment in signal amplitude due to the longer distance between the tripole contacts; or, 2) some of the CAP amplitude is picked up by the reference contacts but this is smaller with ring contacts thus yielding larger potentials when a dot contact records against two ring contacts. Since it has been shown that the effect of longer distances between contacts falls short of balancing against the benefit of the additional short-circuited electrodes [10], the second explanation should thus be considered. The SMES amplitude was on average 32% larger (p<0.05) in the mixed tripole, however, the increment in CAP was even higher with 129% (p<0.05), therefore yielding an improved SNR. It is worth noting, as mentioned before, that the outer tripole contact displacement inwards the cuff did not contribute to a higher SNR in this specific design. The question as to whether an optimal distance to the cuff ends could incline the balance towards the short-circuited tripole configuration has still to be investigated. Our work, however, aimed at investigating whether splitting ring contacts into discrete dots would negatively affect the SNR. Our results suggest that if only the central contact is reduced to a dot, the SNR of the short-circuited tripole would actually improve. This is because on average, the mixed tripole yields an IR(SA) of 100% and IR(SMES) of 87%, when compared with its ring counterpart (p<0.05). Furthermore, Chu *et al.* has recently proposed an additional middle ring contact as an enhancement of the ring tripolar configuration [44]. This “revised-tripole” was incidentally studied in our experiment (+-.-+). SNR improvements for SA and MES were observed in both studies independently. The mixed tripole, however, still produced higher SNR, but more importantly, splitting the central contacts of the revised-tripole was also found beneficial (see Figure 7 and 13).

The selectivity of a discrete contact is conditioned by its distance to the source. In the case where a signal of particular interest is conducted in a fascicle situated far from the dot contact, a higher SNR for that particular signal might be achieved using a ring contact instead. On the other hand, Rozman *et al.* have shown that having sufficient, and shortly spaced contacts (0.5 mm), selection of the best placed electrodes based in SNR is possible [21].

**Figure 14:**
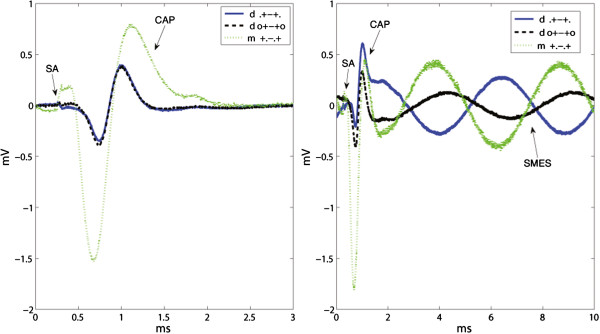
**Recordings obtained using dot, short-circuited dot and mixed tripolar configurations. **Since the “end effect” was found negligible in our experimental setup, the difference in SNR can be mostly attributed to the change between ring and dot outer contacts. The mixed tripole outperforms the others mainly because of the large compound action potential (CAP). The stimulation artifacts (SAs) and CAPs appear chronologically in the left inset without the induced simulated myoelectric signal (SMES). Recordings from all the configurations in each inset were obtained during the same session.

Previous research has shown that moving the contacts inwards the cuff yields a limited improvement [10] which disappears at 5 *mm *according to our results. Therefore, we can argue that the comparison between dot (d.+-+.) and mixed (m+.-.+) tripoles is mainly due to the difference between ring and dot end contacts. These SNR improvements reached around 191% for SMES, and 80% for SA, both statistically significant. This observation suggests that a mixed tripole is preferable to a dot only tripole, even if it includes an additional pair of short-circuited ring contacts. The SNR improvement for the mixed tripole (m+.-.+) and short-circuited dot tripole (do+-+o) was around 96% for SMES, and 48% for SA, both statistically significant. A recording example obtained within the same session in those three configurations is given in Figure 14. The advantage of the mixed tripole, mainly due to the higher CAP amplitude, can be clearly appreciated in this figure.

Reducing only the central ring to a dot contact has favorable practical implications. A smaller contact area is more easily obstructed from the site of recording interest (e.g. if placed over a blood vessel during implantation). When using end ring contacts, this problem is only of concern for the central contacts. Fewer contacts also reduce the number of wires required.

Despite the large values (16% reduction (r/d) for SA, but 67% improvement (r/d) for SMES) the difference in SNR between all ring and all dot montages (.+-+.) remain statistically non-significant. Similar contrasting findings result from the comparison between short-circuited tripoles (o+-+o) with 29% reduction (r/d) and 50% improvement (r/d). These results indicate that the interfering signals are rejected differently depending on the source location and the recording configuration. This is in line with the finding that the best SNR against SA corresponds to the modified mixed tripole (+—+). In any case, the tripole and short-circuited tripole have the best SNR tradeoff for both kinds of noise.

### Nerve conduction velocity and experimental model

The nerve conduction velocity determines the optimal inter-electrode distance for recording CAPs. It is thus obvious that with very long cuffs and increasing distances between contacts, there is a point where the CAP amplitude will no longer increase while the noise figure, independent of the conduction velocity. This is expected to happen at shorter lengths and distances with slow conducting fibers. Here, we use a nerve with a rather slow conduction velocity compared to the later foreseen human applications while long cuffs quickly become surgically unrealistic. The conclusions given hereafter thus seem broadly applicable but it should also be pointed out that in a follow up study, variations of the frog model temperature will be used to fully explore the effects of conduction velocity.

Previous research has mostly resorted to mammal models for investigating the impact of cuff designs on the SNR [10,11,44]. This involves chronic implantation and requires longer times of specialized care. In this work, we have shown that comparable results can be achieved in a simpler *in vitro* model if the aim of the study is solely to investigate IRs on SNRs. The measured effect of an additional pair of short circuited electrodes and the “end effect” are in line with previous findings [10,12,38,39,44], thus reassuring the presented methodology.

## Conclusion

In this work, different cuff electrode contact configurations were explored by comparing recordings obtained with well-established montages, namely the tripole and the short-circuited tripole. Our results show that a mixed tripole (m+.-.+) performs better than a similar configuration including only ring contacts. Therefore, splitting the central ring contact of a cuff electrode into a number of dot contacts, not only results in additional channels to potentially retrieve more information, but more importantly, an improved SNR is obtained with this modification.

This conclusion also holds for the well-known short-circuited tripole, usually considered the best performing montage. The dimensions of the short-circuited tripole used in this study, however, did not allow to take advantage of the “end effect”. Therefore, further research is required to determine the optimal size for short-circuited mixed tripole.

## Competing interests

MOC and RB were partially funded by Integrum AB which is interested in solutions for prosthetic control. This particular interface is one of many to be evaluated through this funding, therefore is in the interest of Integrum AB that the results are impartial and as accurate as possible. No competing interest is linked with the results of this study. JMM, JD and BH declare not to have competing interests.

## Authors’ contributions

MOC and JD designed the experiment. JMM made the *in vitro *preparation. MOC obtained the recordings, analyzed the results and drafted the manuscript. JD, RB and BH supervised this research and revised the manuscript. All authors read and approved the final manuscript.
